# Clinicoetiological Characterization of Infectious Vaginitis amongst Women of Reproductive Age Group from Navi Mumbai, India

**DOI:** 10.1155/2015/817092

**Published:** 2015-08-17

**Authors:** Anuradha Narayankhedkar, Anahita Hodiwala, Arati Mane

**Affiliations:** ^1^Shahbazker's Diagnostics Pvt. Ltd., Mumbai, Maharashtra 400005, India; ^2^Department of Microbiology, MGM Medical College, Navi Mumbai, Maharashtra 410209, India; ^3^Department of Microbiology & Clinical Pathology, National AIDS Research Institute, Pune, Maharashtra 411026, India

## Abstract

Vaginitis is one of the commonest reproductive tract infections in sexually active women. In the present study clinicoetiological characterization of infectious vaginitis amongst 380 women of reproductive age group (18–45 years) was done. Bacterial vaginosis (BV) was detected by Nugent's scoring,* Candida *infection by culture, and trichomoniasis (TV) by wet mount. One hundred and ten (28.9%) women presented with symptoms of vaginitis. The presenting symptoms were vaginal discharge 106 (96.4%), vulval itching/irritation 19 (17.3%), malodor 5 (4.5%), pain in abdomen 3 (2.7%), and dysuria 1 (0.9%). The commonest etiology detected was* Candida *in 33 (30%) cases, of which 18 (54.5%) were* C. albicans *and 15 (45.5%) non-*albicans Candida *(NAC) infections. The NAC isolates were* C. glabrata *(*n* = 10),* C. tropicalis *(*n* = 3), and* C. krusei *(*n* = 2). BV and TV were observed in 19 (17.3%) and 2 (1.8%) cases, respectively. A statistically significant association between* Candida *infection and presence of curdy-white discharge (*p* = 0.001) and vulval itching/irritation (*p* = 0.007) was noted. To conclude, we observed the etiological predominance of* Candida *infection, with considerable prevalence of NAC, indicating the need for microbiological investigation up to species level in cases of* Candida *infections, to ensure appropriate management.

## 1. Introduction

Reproductive tract infections (RTI) including sexually transmitted infections (STI) present a huge disease burden and adversely impact the reproductive health. Their consequences are far more devastating and widespread among women as compared to men [1, 2]. Vaginitis is one of the commonest RTI and is characterized by vaginal discharge, vulvar itching/irritation, and malodor. It encompasses three main etiologies, namely, bacterial vaginosis (BV), vaginal candidiasis (VVC), and trichomoniasis (TV), which generally account for 90% of all etiologies.

BV is the most common cause of vaginal discharge among women in reproductive age. It is associated with an increased vaginal pH and replacement of vaginal* Lactobacilli* with* Gardnerella vaginalis* and anaerobic Gram-negative rods [3]. BV is of special public health concern because of the high burden of reproductive and pregnancy related morbidity [4]. Likewise high coinfection rates with other STI raise the possibility that BV increases susceptibility to STI [4, 5]. VVC affects up to 75% of reproductive age women at least once during lifetime. Though majority of the infections are caused by* Candida albicans* the incidence of non-*albicans Candida* (NAC) infection is being increasingly reported nowadays probably due to low dosage azole maintenance regimens and the use of over-the-counter antimycotics [6].* Trichomonas vaginalis* infection is the most common curable STI worldwide. Trichomoniasis is associated with infertility, enhanced predisposition to neoplastic transformation in cervical tissues, and an increased risk of transmission of other STI, including* human immunodeficiency virus* (HIV), by as much as twofold [7, 8].

The symptoms of vaginitis are nonspecific and diagnosis without laboratory confirmation can lead to inappropriate medication [6]. Furthermore the etiological profile varies from country to country and from one region to another within the same country. The present study was undertaken for the clinicoetiological characterization of infectious vaginitis amongst women of reproductive age group attending the outpatient clinic of Obstetrics and Gynaecology Department from Navi Mumbai, India.

## 2. Materials and Methods

The cross-sectional observational study was conducted in the Department of Microbiology in association with the Department of Obstetrics and Gynaecology at MGM Medical College and Hospital, which is a referral and teaching hospital located in Kamothe, Navi Mumbai, India. A part of the study was carried out at National AIDS Research Institute, Pune. The study was approved by the institutional Ethics Committee of the MGM Medical College and Hospital.

A total of 380 women attending the clinic from July to December 2014 were screened for symptoms of vaginitis. The inclusion criteria were women with age between 18 and 45 years and presenting with symptoms of vaginal discharge, vulval itching/irritation, and/or vaginal malodor. Women who were pregnant, who were menstruating, who had received antibiotics in the past 4 weeks, and who were not consenting to participate were excluded.

### 2.1. Specimen Collection and Processing

Informed written consent was taken from patient before enrolment in the study, followed by detailed physical examination of the patient. Per speculum examination was performed and the vaginal mucosa was inspected for presence of erythema, lesions, and discharge. Vaginal material was collected from the lateral vaginal wall with cotton-swab. Three swabs per patient were collected.

The first swab was used to determine the vaginal pH by touching a pH strip and comparing the change against a reference reader; it was then smeared on to a glass slide for Gram staining and finally 2 drops of 10% potassium hydroxide were added to it to determine amine/fishy odour (whiff test). The Gram stained slides were evaluated using Nugent's scoring system for detection of BV [9]. A Nugent score of 0–3 was interpreted as negative for BV and a score of 4–6 as intermediate while a score of 7–10 was interpreted as consistent with BV.

The second swab was placed in screw-cap plastic tubes containing 0.5 mL of 0.9% saline to carry out wet mount microscopy for detection of TV. Swab was vigorously rotated in the saline and pressed against the side of the tube to express as much fluid as possible. One drop of the expressed fluid was placed on glass slide with a cover slip and examined at magnification of 200x within 15 minutes of collection of the sample. The positive result was defined as the presence of one or more Trichomonads with characteristic morphology and jerky motility [10].

The third swab was used for* Candida* culture on Sabouraud's dextrose agar followed by incubation at 37°C for 48 h.* Candida* isolates were identified by the standard mycological techniques like cultural characteristics, Gram stain, germ tube formation, and the biochemical profile on API 20 C AUX (BioMerieux, France) [11]. All cases with* Candida* as the etiological agent also had a clinical diagnosis of VVC.

### 2.2. Statistical Analysis

The GraphPad statistical software (GraphPad Software Inc., USA) was used for statistical analysis. Descriptive analysis was performed for all variables and data presented as percentages. The Chi-square or Fisher's exact test as applicable was applied for analysis of categorical variables. A *p* value of <0.05 was considered to be statistically significant.

## 3. Results

Of the 380 women screened, 110 (28.9%) had symptoms of vaginitis. The prevalence of infectious etiologies, namely, bacterial vaginosis, candidiasis, and trichomoniasis, was determined in these women. The median age of the women was 28 years (interquartile range, 24–32); all of them were married and cohabiting with their husbands, while 13 (11.8%) were nulliparous, 48 (43.6%) primiparous, and the rest multiparous.

The presenting symptoms were vaginal discharge 106 (96.4%), vulval itching/irritation 19 (17.3%), malodor 5 (4.5%), pain in abdomen 3 (2.7%), and dysuria 1 (0.9%).

The attributes of vaginal discharge are presented in Table 1.

**Figure 1:**
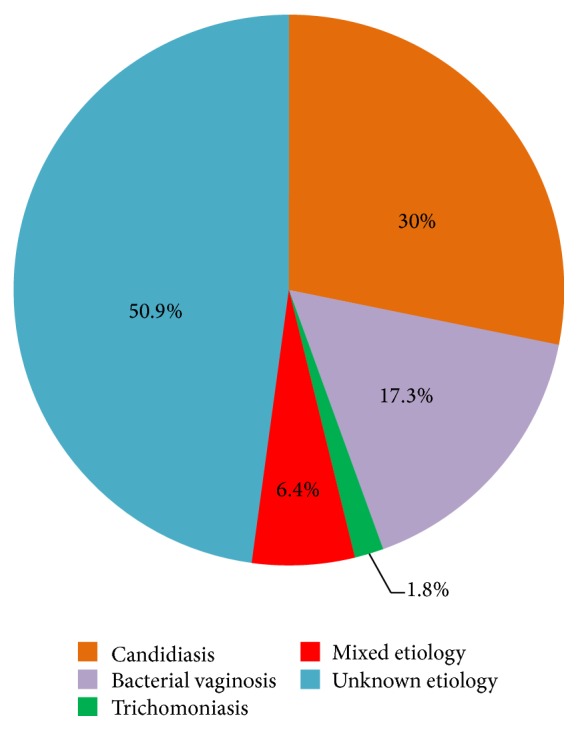
Distribution of etiologies of infectious vaginitis.

In the 110 women presenting with vaginitis, 54 (49.1%) infectious etiologies were identified. The etiological agent was identified in 47 (42.7%) women, of which 7 (14.9%) had mixed etiologies.  Figure 1 shows the etiological distribution in infectious vaginitis.

The commonest etiology was candidiasis detected in 33/110 (30%) cases.* C. albicans* was isolated in 18/33 (54.5%) cases, while 15/33 (45.5%) cases had NAC isolates. The NAC isolated were* C. glabrata* (*n* = 10),* C. tropicalis* (*n* = 3), and* C. krusei* (*n* = 2).

BV (Nugent's score 7–10) was observed in 19/110 (17.3%) cases. TV was detected in 2/110 (1.8%) cases. Mixed etiologies detected were BV + VVC in six cases and BV + TV in one case.

We observed a trend of decrease in the prevalence of infectious vaginitis with age as presented in Table 2; however the difference was not statistically significant between the different age groups (*p* = 0.187).

The association between clinical symptoms and microbiological diagnosis is presented in Table 3. A statistically significant association between candidal infection and the presence of curdy-white discharge (0.001) and vulval itching/irritation (*p* = 0.007) was noted.

## 4. Discussion

Vaginitis is the commonest RTI in sexually active women and is associated with a significant risk of morbidity. The management of vaginitis remains largely empirical, though establishing correct diagnosis is the most important factor for successful treatment [12]. Variable prevalence rates of infectious vaginitis, attributable to the varied etiologies studied, the detection techniques applied, patient groups involved, and the geographical locales have been reported by studies conducted globally as well as in India [13–18]. In the present study majority of symptomatic women had unidentified etiology. This finding requires due consideration and indicates the need for looking into other etiological agents including but not limited to gonorrhea, chlamydiasis, viruses that may lead to vaginitis.

VVC was the commonest infectious etiology identified in the present study and its prevalence was consistent with earlier reports [13, 19–24]. The distribution of* Candida* spp. identified in women with VVC varies widely depending on the locations as well as the populations studied [25]. Recently a number of studies have shown an increasing trend of NAC infection [20, 21, 26]. Though* C. albicans* remained the predominant species isolated in the present study, NAC were isolated in considerable proportions. Furthermore in accordance with previous literature* C. glabrata* was the commonest NAC isolated [21, 27]. With higher resistance levels of most NAC species to the commonly prescribed azole-based treatments, the consequences for women affected by these strains might be incapacitating. Hence microbiological investigation up to species level should be made mandatory for* Candida* infections to ensure appropriate management.

The prevalence of bacterial vaginosis in present study is 17.3%. Various studies show prevalence of BV ranges from 2.5% to 48% [14, 17, 28–33]. These variations may be because of differences in study population, economic status, educational background, and method used for detection of bacterial vaginosis [30]. The prevalence of BV in the present study is in accordance with other reports from India [4, 29]. It is noteworthy that the mixed microbial etiologies observed were all seen in association with BV. It may thus be speculated that women with BV tend to lose natural protection against genital tract infections leading to acquisition of coinfections like* T. vaginalis* and* Candida.* The mechanisms underlying these relationships are not well understood and warrant further investigation [4].

Relatively lower prevalence of trichomoniasis (1.8%) was observed in our study, a finding consistent with researchers' earlier reports [14, 15, 18, 29, 30, 34]. On the contrary, a number of studies have reported a higher prevalence of TV than ours [35–38]. These differences could be due to variation in personal hygiene practice, environment, and socioeconomic and cultural factors of the study participants [14]. Moreover, the detection of TV by conventional wet mount method in the present study might have reduced the actual prevalence.

To conclude, we present here the clinical and etiological characterization of infectious vaginitis amongst women of reproductive age group from Navi Mumbai, India. We observed the etiological predominance of* Candida* infection, with considerable prevalence of NAC, indicating the need for microbiological investigation up to species level in cases of* Candida* infections, to ensure appropriate management.

## Figures and Tables

**Table 1:** Attributes of vaginal discharge.

Attribute	Number *N* = 106	Percentage
Color		
White	87	82.1
White curdy	15	14.2
Greenish	1	0.9
Creamish	3	2.8

Quantity		
Scanty	26	24.5
Moderate to copious	80	75.5

Consistency		
Thin	43	40.6
Thick	63	59.4

Duration		
≥15 days	30	28.3
<15 days	76	71.7

pH		
4	54	50.9
5	37	34.9
>5	15	14.2

Malodor		
Present	5	4.7
Absent	101	95.3

**Table 2:** Age-wise distribution of vaginitis.

Age group	Infectious vaginitis *N* (%)	Bacterial vaginosis *N* (%)	Candidiasis *N* (%)
18 to ≤24 years	24 (44.4)	10 (52.6)	16 (48.5)
25 to ≤34 years	21 (38.9)	6 (31.6)	13 (39.4)
35 to 45 years	9 (16.7)	3 (15.8)	4 (12.1)

**Table 3:** The association between clinical symptoms and microbiological diagnosis.

	Candidiasis		Bacterial vaginosis	
	*n* = 33		*n* = 19	
	Present	*p*	Present	*p*
	*N* (%)		*N* (%)	
Vaginal discharge *n* = 106	32 (30.2)	1.00	17 (16.03)	0.137
Curdy-white discharge *n* = 15	13 (86.7)	**0.001**	1 (6.7)	0.297
Vulval itching/irritation *n* = 19	12 (63.2)	**0.007**	4 (21.1)	0.519
Pain in abdomen *n* = 3	—	0.552	2 (66.7)	0.076
Malodor *n* = 5	1 (25)	1.000	2 (40)	0.205
Dysuria *n* = 1	—	1.000	1 (100)	0.172

The group of women with candidiasis and bacterial vaginosis were compared with the respective group of women without infection. Trichomoniasis was not included as there were only 2 cases.
